# Food-Evoked Emotion, Product Acceptance, Food Preference, Food Choice and Consumption: Some New Perspectives

**DOI:** 10.3390/foods12112095

**Published:** 2023-05-23

**Authors:** Witoon Prinyawiwatkul

**Affiliations:** School of Nutrition and Food Sciences, Louisiana State University, Agricultural Center, Baton Rouge, LA 70803, USA; wprinya@lsu.edu; Tel.: +1-225-578-5188

## Introduction

Food is more than just a source of nutrients—it is a source of basic pleasure and aesthetic experiences. Consumers are now more health-conscious and more educated about what goes into their foods. Many consumers want healthier versions of retailed food products, such as those with high-fiber or low-sodium content. These consumers are concerned about the health benefit or risk associated with food consumption. Currently, globalization allows consumers to be exposed to various cuisines which can be readily available to them. Additionally, with the world population increasing rapidly, alternative food sources and food production will be needed to support sustainability. Potential alternative protein sources (e.g., from edible insects or plant-based sources), for instance, will likely be needed. By-products from various food processing and manufacturing find their way to be incorporated into food and beverage with additional health benefits. The questions are “would consumers be willing to consume them? How do they feel when consuming them, and do they like them?”. Based on many studies, food choice, acceptance, preference, and consumption are affected by many factors, including both intrinsic and extrinsic factors and cues and consumer characteristics.

It is known that food elicits emotions, mainly positive ones. Measuring food-evoked emotions is a topical topic in sensory and consumer sciences. New methods for emotion measurement have been proposed. Emotions are becoming a critical component in designing products that meet consumers’ needs and expectations [1]. Emotional profiles and ratings may effectively differentiate products with similar sensory characteristics and hedonic ratings; hence, they may provide additional information that goes beyond traditional hedonic ratings and may provide more insight toward food choice and behavior. Several studies have reported emotional responses to food and their relationships to product acceptability. In addition to the sensory quality of food, food-evoked emotion has been reported to be critical in predicting product acceptance and willingness-to-try, which are, in turn, critical in developing novel products. Appropriate health benefit information has also been reported to impact emotion, purchase decisions, and food choices. Human senses and cues perform an instrumental role in food choice and intake, emotion, and product acceptance; hence, understanding their roles and importance is critical.

This Special Issue aimed to present both original and cutting-edge research contributing to a deeper understanding of food-evoked emotion, food choice, preference, acceptance, and consumption, which is a valuable source of information for research and development, product innovation, and marketing that goes beyond traditional sensory preference and acceptability measurement. In this Special Issue, there are eighteen papers showcasing a diversity of approaches applied to different types of food products as test samples that provide excellent examples of the complex and multidisciplinary nature of the subject matter. A snapshot of these papers is given below.

Several methods and testing conditions for measuring and collecting emotions associated with foods have been developed and reported. Several studies reported in this Special Issue provide additional insights in this area. Different questionnaire designs can influence the results of emotion and wellness assessments. Hanmontree et al. assessed emotion and wellness profiles of herbal drinks using six different questionnaire designs [a questionnaire item written in the form of words vs. full sentences and three types of measuring methods: a rating scale, CATA, and RATA] [2]. They reported that using a full sentence did not provide a clear benefit over using some keywords, especially when using familiar words clearly understood by consumers. All three measuring methods produced similar emotion and wellness profiles. However, the authors cautioned that each method has advantages and limitations that should be carefully considered [2]. For emotion measurement, questionnaires are primarily used and can be implemented using words or emojis. However, it has been questionable if emojis are appropriate to be used in the context of food consumption situations. Jeager et al. used 24 emojis (14 facial and 10 non-facial) characterized using the Pleasure-Arousal-Dominance model and compared the perception of two consumer groups (*n* = 165 from New Zealand and *n* = 861 from the UK) [3]. They found that emojis were suitable for cross-cultural research and identified similar groups of emoji as most and least suitable for food-related consumer research [3]. In order to be effective in collecting, sorting, and arranging a large pool of data on consumers’ perceptions, Chen et al. introduced novel approaches—text mining and Natural Language Processing (NLP)—to obtain sensory data and identify current consumer trends on alternative proteins [4]. Based on some selected publications between 2018 to 2021, they found that insect- and plant-based proteins were the centers of alternative protein research, and there was no significant association between emotions and alternative protein categories [4].

Several studies have reported that additional product information, along with relevant information, such as health benefits and sustainability, can significantly impact consumers’ perceptions. Gurdian et al. [5,6] investigated the effects of ingredient (that is, cricket protein) information, environmental and nutritional-quality claims, and tasting moments [before tasting and after tasting with or without additional statement) on hedonic, emotional, and purchase intent (PI) of pita chips and chocolate brownies. Although expectations, disconfirmations, and product claims may not have a direct effect on product liking, they may still indirectly affect the overall product acceptability via emotional elicitation [5]. Evoking “interested” and “adventurous” emotions performed a significant role as drivers of product liking regardless of the formulation and moment, and PI can be improved through the elicitation of pleasant emotions upon tasting. For chocolate brownies, the disclosed information affected emotional profiles more than formulation. After-tasting emotions “happy” and “satisfied” were critical predictors of PI. Gender also significantly affected perceived emotion and PI [6]. Another study was devoted to understanding how the “green-food label” of rice affected the perception and emotions of Chinese consumers using a text mining approach [7]. Green food is produced in an ecological environment that meets standards of safety, nutrition, and non-pollution. The COVID-19 pandemic increased public health awareness, turning consumers toward safe and healthy foods to strengthen their immunity. Xu et al. compared differences in consumer perceptions and emotions towards green-labeled rice vs. conventional rice [7]. They reported that green-label mistrust and packaging breakage during logistics were the leading causes of negative emotions (e.g., disappointing and doubtful) among consumers of green-labeled rice [7].

Environments, where consumers make food choice decisions and consume food, have effects on their consumption, choices, preference, and product acceptance. This may likely and partly due to different emotional responses induced by repeated exposure to such environments. Vanhatalo et al. designed two environments in the restaurant: ‘nature ambiance’ to induce a positive emotional response and ‘fast food ambiance’ to induce a less positive emotional response [8]. They found that during ‘nature ambiance’ week, consumers more often chose vegetarian dishes and generated less plate waste. This study provided some evidence that ambiance modification in a real-life setting may insert a moderate effect on healthier and more sustainable food behaviors [8]. In another study, Tsai and Chen focused on the “trust” level of older adults that influenced continuance intention in congregate meal halls [9]. Trust is a mental state and a basis of social participation. In this study, trust was classified as “pre-use trust” and “post-use trust” in products or services. The authors found that the perceived service quality was the main factor affecting the perceived satisfaction, which, in turn, affected the post-use trust; hence, the older adults showed a positive continuance intention to participate in the senior meal halls [9].

In addition to methods and testing conditions and environments as described above, the type of test products and consumer characteristics can largely affect acceptance, emotion, food choice, and consumption. Insects have been proposed as a sustainable and alternative food source; however, in the Western world, insects are viewed as disgusting. Serpico et al. attempted to understand the acceptance of insect-based products in the US market by studying emotional responses [10]. Insect food products were positively correlated with the emotions of interest, understanding, daring, adventurous, and worried and negatively correlated with the emotions satisfied, good, pleasant, happiness, calm, warm, nostalgia, and secure. Disgust was found as a barrier to product acceptance [10]. Likewise, Penedo et al. found that the most common reason among young adults (*n* = 290 in a Swiss university) for not eating insect-containing foods was disgust. In contrast, the most likely reason for eating insect foods was curiosity [11]. Product appropriateness for insect incorporation performs a significant role in the acceptance of insect-containing food. Ho et al. evaluated how product eating experience affected consumer acceptability, emotional response, satiety, and plate waste [12]. Three different dishes (sausage, pasta, and brownies) containing cricket powder (CP) were tested against the controls. Negative terms selected, such as worried, decreased once the products were consumed. Significant correlations were found between appearance liking and satiety and taste liking and plate waste for both the control and CP dishes. They concluded that formulation and serving methods might increase the overall acceptability of food products containing insect powders [12].

Two studies were devoted to evaluating consumer perception of products containing byproducts from seafood [13] and brewing [14] processing. With increasing global demand for seafood, seafood byproducts (SB) utilization can contribute to a more sustainable food supply chain through waste-to-value food product development. Murillo et al. evaluated some factors influencing consumers’ willingness-to-try (WTT) seafood byproducts using an online survey with 904 adult seafood consumers [13]. This research was concerned with using consumers’ emotional baseline scores during an early stage of the COVID-19 pandemic as predictors of WTT foods containing SB. Consumers feeling more unsafe during the pandemic may have been reluctant to report positive WTT. The SB safety and health claim information cue may have reassured some of these consumers, making “unsafe” emotion a significant predictor of WTT [13]. Brewery spent grain (BSG) is a byproduct from a brewing process of malted barley and a good source of fiber (30–50% dry wt basis). Curutchet et al. evaluated the impact of this fiber enrichment on sensory quality, acceptability, and purchase intention of bread, pasta, and chocolate milk [14]. Ambivalence was seen in the emotions generated by the different fiber-enriched products, depending on whether the fiber was perceived. Consumers felt more warm, bored, active, and less guilty when tasting fiber-enriched pasta and bread, but the opposite was observed for fiber-enriched chocolate milk. The authors concluded that the effect of BSG addition was product-specific and that fiber perception makes consumers feel more confident [14].

Cardello et al. conducted a survey of willingness to consume (WTC) 5 types of plant-based (PB) (Milk, Cheese, Meat (33%PB), Meat (100%PB) and Fish) food in the USA, Australia, Singapore, and India (*n* = 2494) [15]. In addition to WTC, emotional, conceptual, and situational use characterizations were also evaluated and found to exert significant lifts/penalties on WTC. They reported that positive valence (‘enthusiastic’, ‘comforting’, ‘easygoing’, ‘energetic’, ‘happy’, and ‘inspiring’) significantly impacted positive WTC, while the five terms with negative valence—‘boring’, ‘dissatisfied’, ‘nervous’, ‘tense’, and ‘uninspired’—overwhelmingly reduced WTC. This study demonstrated that consumers are not monolithic in their WTC PB foods and that WTC is often a function of the food category of the PB food [15].

Coffee is one of the most widely consumed beverages in the world, and black coffee drinking is becoming more popular and a new cultural practice among Thai people. The link between coffee aroma/flavor and elicited emotions remains underexplored. Pinsuwan et al. identified key sensory characteristics of brewed black coffee via descriptive sensory analysis that affected acceptance, purchase intent, and emotions for Thai consumers [16]. This information could be useful for creating or modifying the sensory profile of brewed black coffee to increase consumer acceptance [16].

Three papers were devoted to product development and sensory strategies for functional and healthy foods. Vrgović et al. described a systematic process so-called “co-creation” for developing functional food, which directly involves consumers in various stages of the creation process [17]. They used raspberry seeds as a source of natural bioactive compounds to produce healthy, functional food products other than just an extract. Montoya et al. developed and characterized reduced-fat pork and chicken meatballs. Inulin was used as a fat substitute to mimic the properties of fat [18]. There was a greater probability of meatball consumption with a claim of “preservative-free” as compared to other claims “reduced-fat” or “a good source of fiber”. Last but not least important, Spence noted the importance of sodium in the human diet while emphasizing the risk of excessive sodium intake [19]. The author provided a narrative historical review, discussing a wide range of sensory approaches that have been experimented with to modulate expected and perceived saltiness in order to reduce salt consumption. The author also highlighted a number of important questions that remains for future research [19].

The editor hopes that the readers will find this Special Issue insightful, interesting, and useful for future research. The diversity of both the content and the methodologies presented in this Special Issue should inspire and encourage future exploration of multidisciplinary research collaboration, which would lead to a better understanding of the complex relationship among emotion, acceptance, preference, choice, and consumption of food.
